# Evaluating the extent of acute radiofrequency ablation lesions in the heart using an inversion recovery SSFP sequence

**DOI:** 10.1186/1532-429X-15-S1-O17

**Published:** 2013-01-30

**Authors:** Haydar Celik, Venkat Ramanan, Jennifer Barry, Sudip Ghate, Vivian Leber, Mohammed Shurrab, Samuel Oduneye, Nilesh R Ghugre, Eugene Crystal, Graham Wright

**Affiliations:** 1 Imaging Research, Sunnybrook Research Institute, Toronto, ON, Canada

## Background

Current MRI methods for radiofrequency ablation (RFA) visualization have problems in accurately delineating the extent of lesions at an early phase. The aim of this study is to evaluate a non-gadolinium enhanced (NGE) multi-contrast inversion recovery steady state free precession (IR-SSFP) imaging method [1] to visualize acute ablation lesions.

## Methods

15 lesions were created in the endocardium of 13 pigs using approved animal protocols. NGE IR-SSFP and T2-w black-blood (double IR-FSE) images were acquired in <60min after ablation. Then, Gd-DTPA (Magnevist, 0.2 mmol/kg) was injected and LGE images were acquired repeatedly over one hour. Gross pathology was used as the reference for lesion size measurements. Two regions were measured in this reference: the pale "inner" lesion core and the "outer" lesion border including the dark rim on pathology (see Results).

## Results

**Figure 1:**
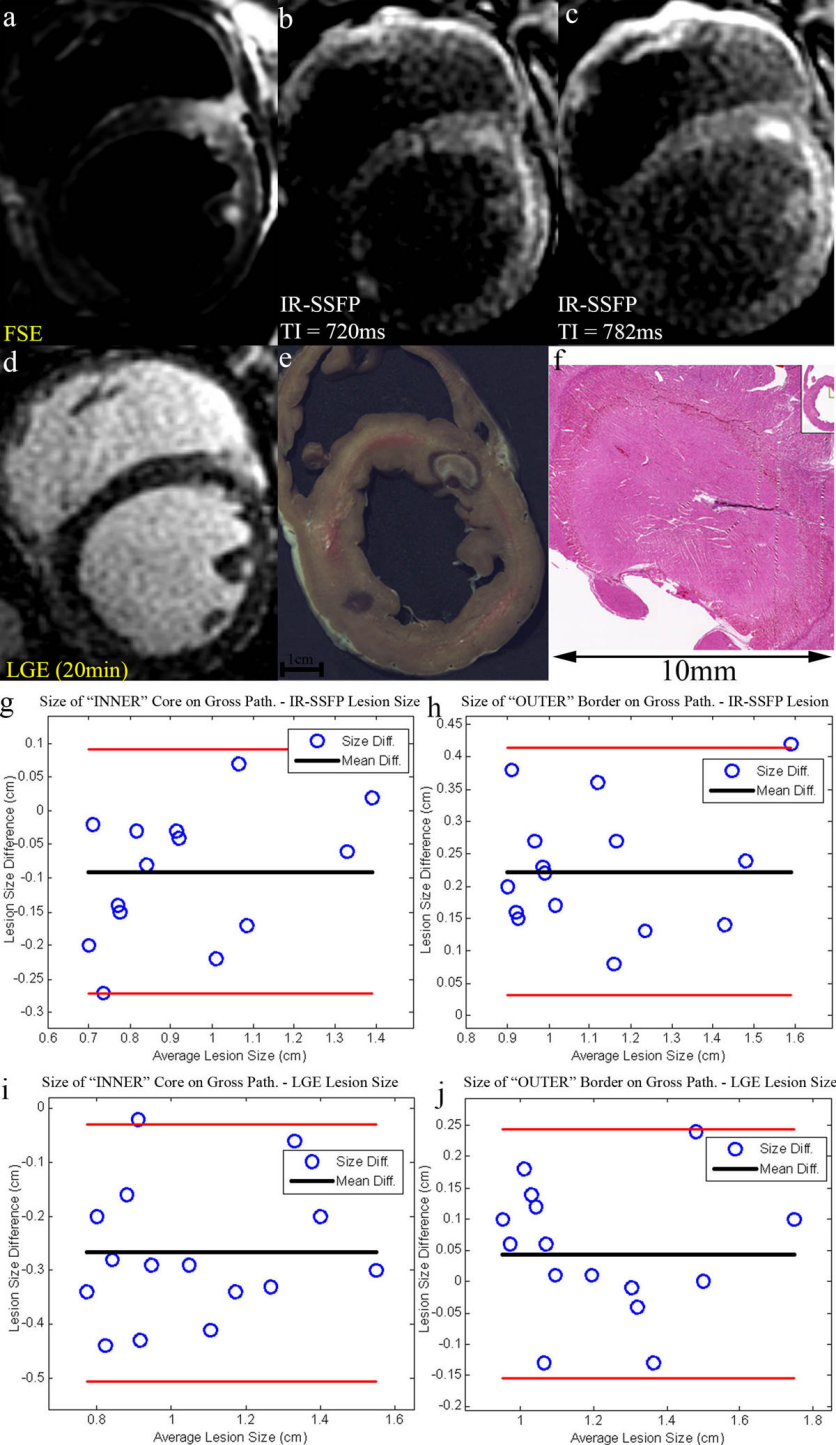
(a) double IR-FSE, (b-c) IR-SSFP, (d) LGE (TI=500ms), (e) gross pathology, (f) H&E stained histology images zoomed in on lesion ; (g) lesion core size and IR-SSFP comparison, (h) outer lesion border size and IR-SSFP comparison, (i) lesion core size and LGE comparison, (j) outer lesion border size and LGE comparison. Red lines indicate 95% confidence limits.

All DIR-FSE images showed large hyper-intense regions in and around the lesion likely due to edema, making, the lesion borders hard to distinguish (Fig 1a). The lesion borders were sharply delineated with the multi-contrast NGE-IR-SSFP sequence (Fig 1b-c) [2]. The size of the lesions as seen on LGE images depended on the time after injection. At early time points the lesions appeared as non-enhanced regions (Fig 1d, (t=20min after Gd-DTPA injection), but later, Gd-DTPA started to enter the lesion border and a bright rim became visible. Therefore, lesion size measurement of the LGE images depends on the time between the injection and imaging and whether the bright rim is included. Here, the lesion size was defined as the combination of the bright rim and dark core in LGE images. Gross pathology showed two distinct areas, the dark rim and the pale lesion (Fig 1e). The inner core showed thermal coagulation and the rim consisted of hemorrhage (Fig 1e), confirmed using histology (Fig 1f).

The lesion sizes measured in LGE images were always larger than those in IR-SSFP images. While the lesion sizes in IR-SSFP images were better correlated with the size of the inner lesion core on gross pathology (Fig 1g), the lesion sizes in LGE images were highly correlated with size of the "outer" lesion border (Fig 1j). Yet to be determined is whether either of these borders corresponds to permanent lesion extent in chronic studies. However, it is clear that double IR-FSE images do not provide reliable data for size measurements.

## Conclusions

IR-SSFP images without Gd enhancement demonstrated good contrast between the ablation lesions and normal myocardium. The lesion size from IR-SSFP images also correlated well with lesion size in gross pathology. Among the imaging methods used in this study, IR-SSFP provided the most reliable and consistent data for RFA lesion characterization.

## Funding

We gratefully acknowledge support from GE Healthcare, the Ontario Research Fund, and Canadian Institutes of Health Research.
